# Integrated Approaches to Drug Discovery for Oxidative Stress-Related Retinal Diseases

**DOI:** 10.1155/2016/2370252

**Published:** 2016-12-07

**Authors:** Yuhei Nishimura, Hideaki Hara

**Affiliations:** ^1^Department of Molecular and Cellular Pharmacology, Pharmacogenomics and Pharmacoinformatics, Mie University Graduate School of Medicine, Tsu, Mie 514-8507, Japan; ^2^Molecular Pharmacology, Department of Biofunctional Evaluation, Gifu Pharmaceutical University, Gifu 501-1196, Japan

## Abstract

Excessive oxidative stress induces dysregulation of functional networks in the retina, resulting in retinal diseases such as glaucoma, age-related macular degeneration, and diabetic retinopathy. Although various therapies have been developed to reduce oxidative stress in retinal diseases, most have failed to show efficacy in clinical trials. This may be due to oversimplification of target selection for such a complex network as oxidative stress. Recent advances in high-throughput technologies have facilitated the collection of multilevel omics data, which has driven growth in public databases and in the development of bioinformatics tools. Integration of the knowledge gained from omics databases can be used to generate disease-related biological networks and to identify potential therapeutic targets within the networks. Here, we provide an overview of integrative approaches in the drug discovery process and provide simple examples of how the approaches can be exploited to identify oxidative stress-related targets for retinal diseases.

## 1. Introduction

The retina is exposed to chronic oxidative stress (OS) through several mechanisms, including constant exposure to light and reactive oxygen species generated by visual signal transduction pathways [1]. In the healthy state, all cell types in the retina are able to maintain homeostasis under conditions of OS [2]. However, when the balance between pro- and antioxidative signaling is compromised, excessive OS induces dysregulation of functional networks and deleterious changes that result in various retinal diseases, including glaucoma [3–5], age-related macular degeneration (AMD) [6, 7], diabetic retinopathy [8], and retinitis pigmentosa [9]. Due to a combination of lifestyle changes and extended life expectancy, an increasing number of people are at risk for these retinal diseases, and the resulting economic burden imposed on health care systems is increasing accordingly. Various therapies have been developed for retinal diseases [10] with some success, most notably the prostaglandin analogs for glaucoma [11] and antivascular endothelial growth factor (anti-VEGF) agents for AMD [12] and diabetic retinopathy [13]. However, there has been a chronic lack of innovation in drug discovery for retinal diseases [14], as there has been for other diseases [15]. For example, therapies targeting OS in retinal diseases have failed to show efficacy in clinical trials; examples are an antioxidant supplement mixture [16] and a hydroxylamine with antioxidant properties [17]. Thus, there is a clear need for novel approaches to the drug discovery process [18–25]. Here, we first provide an overview of some emerging integrative approaches to therapeutic target discovery and then provide some examples as they relate to OS in retinal diseases.

## 2. Integrative Approaches in the Search for Therapeutic Targets for OS in Retinal Diseases

### 2.1. Overview of the Integrative Approaches

**Figure 1:**
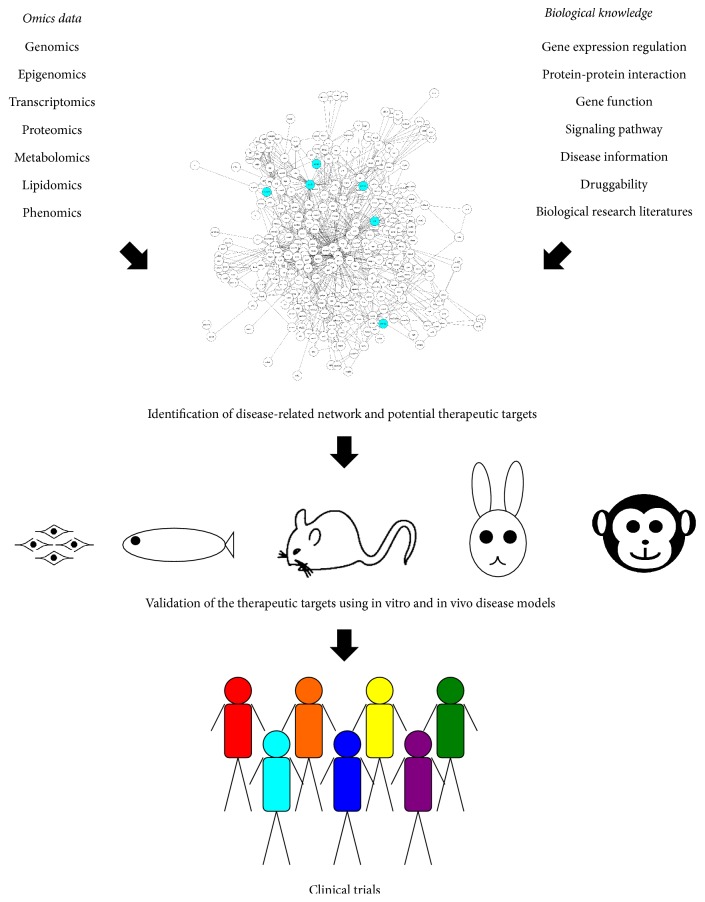
Integrative approaches to identify therapeutic targets. Integration of multilevel omics data and biological knowledge allows construction of disease-related networks and the discovery of potential therapeutic targets (represented as cyan circles in the network). In vitro and in vivo disease models can be used to validate the therapeutic targets and drugs, and drugs displaying efficacy in preclinical models can then be moved into clinical trials.

Improving our understanding of disease pathogenesis is an important step in the identification of therapeutic targets [14]. Recent technological advances have enabled us to obtain large amounts of multilevel omics data [20, 21]. For example, DNA microarray and next-generation sequencing technologies have made it relatively easy to obtain genomics data, including profiling of single nucleotide polymorphisms, copy number variations (CNVs), and mutations, which can be used for genome-wide association studies (GWAS) to identify diseases associated with these changes. The same technologies have facilitated the collection of epigenomics data, such as profiles of DNA methylation and DNA-binding sites, and of transcriptomics data, such as messenger and noncoding RNA profiles. In addition, advanced mass spectrometry-based technologies have been indispensable for profiling of protein expression and protein-protein interactions (proteomics), intermediary metabolites (metabolomics), and lipids (lipidomics) [26, 27]. The more recently developed clustered regularly interspaced short palindromic repeats-Cas9 (CRISPR-Cas9) technology has revolutionized the approach to large-scale loss-of-gene-function experiments and phenotypic analysis (phenomics) at both the in vitro and the in vivo levels [28, 29]. In turn, the need to distill these multilevel omics datasets into biological knowledge has led to equally important advances in data curation supported by public databases and novel bioinformatics tools. Some examples are ENCODE for gene expression regulation [30], STRING for protein-protein interactions [31], gene ontology for gene function [32], KEGG for signaling pathways [33], OMIM for human diseases [34], and DrugBank for chemical structures and drug targets [35]. The biological research literature, in combination with text-mining tools such as Agilent Literature Search [36], represents a rich and ever-expanding source of biological knowledge [37]. Integration of multiomics data with existing biological knowledge is essential for generating accurate disease-related networks and for identifying potential therapeutic targets (Figure 1). For example, a recent meta-analysis of omics data identified a number of approved drugs that could potentially be repurposed for the treatment of rheumatoid arthritis (RA) [36]. In that study, RA-associated genes identified by GWAS were prioritized based on eight criteria, including expression quantitative trait loci, protein-protein interactions, pathway analysis, and text mining. This analysis revealed that targets of approved therapies for RA and other indications were significantly enriched in the prioritized genes [38]. Such network-based frameworks generated by integration of multilevel omics data and biological knowledge can be extended to address numerous problems, including interpretation of GWAS data, identification of disease modules located near the drug target, and discovery of disease-disease relationships [39].

Nevertheless, the potential therapeutic targets identified by such integrated approaches will still need to be validated using in vitro and in vivo disease models. Three-dimensional retinal cultures derived from human or mouse embryonic stem cells and induced pluripotent stem cells have been developed and can be used to validate the therapeutic targets for retinal diseases [40–42]. Advances in genome-editing technologies such as transcription activator-like effector nucleases (TALEN) and CRISPR-Cas9 have made it possible to knock out any gene of interest in various species, including teleosts such as zebrafish [43–47]. Indeed, a number of retinal disease models have been developed in zebrafish [48–55], rodents such as mouse and rat, lagomorphs such as rabbit [56–59], and primates such as the common marmoset and cynomolgus monkey [60–62]. Such models have been used successfully to validate the efficacy of therapeutic drugs for retinal diseases, allowing them to be moved forward into clinical trials (Figure 1).

### 2.2. An Integrative Approach towards Drug Discovery for Glaucoma-Related OS

**Figure 2:**
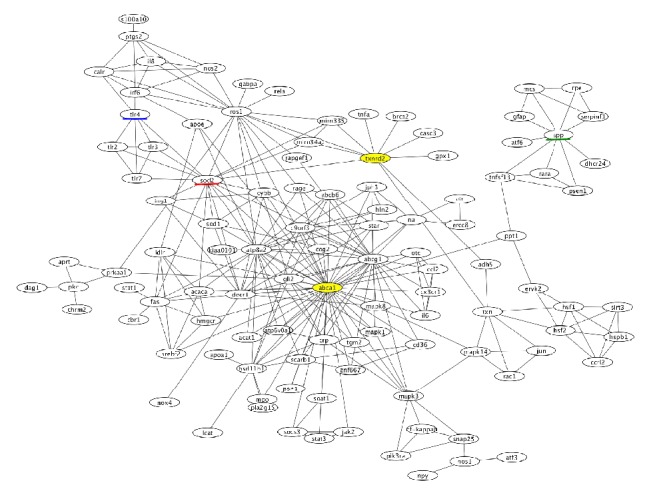
A network related to oxidative stress in glaucoma. An Agilent Literature Search was used as the basis for the network. We used 39 glaucoma-associated genes identified by GWAS as the target molecules and “oxidative stress” as the designated context in the Agilent Literature Search. The glaucoma-associated genes in the network are shown in yellow and other potential therapeutic targets are underlined.

To illustrate how this integrative approach can be used for OS in retinal diseases, we examined (i) whether glaucoma-associated genes identified by GWAS might be connected through genes related to OS and, if so, (ii) whether the network could identify therapeutic targets to reduce OS in glaucoma. To this end, we used Agilent Literature Search [36], a literature mining tool that can extract biological associations related to a target entity (e.g., gene, mRNA, protein, molecule, chemical, drug, and disease) in a particular context from biomedical literature databases such as OMIM [34] and PubMed [63]. The relationships identified through this analysis (e.g., in our case, glaucoma-associated gene X induces the expression of gene A through interaction with gene B under conditions of OS) can be represented as a network(s) using Cytoscape [64]. If the network of glaucoma-associated gene X and that of glaucoma-associated gene Y share a node(s), these networks can be connected through the node(s), resulting in an integrated network. We used 39 glaucoma-associated genes identified by GWAS [65–67] as the target entities and “oxidative stress” as the designated context in an Agilent Literature Search. The resulting network, shown in Figure 2, contains two glaucoma-associated genes: ATP-binding cassette subfamily A member 1 (ABCA1) and thioredoxin reductase 2 (TXNRD2) (shown in yellow in Figure 2). Gene ontology analysis using DAVID [68] revealed that genes related to “response to oxidative stress” are significantly (*p* = 2.5 × 10^−10^) enriched in the network, suggesting that ABCA1 and TXNRD2 are connected through genes related to OS. Superoxide dismutase 2 (SOD2, underlined in red in Figure 2) is another gene related to “response to oxidative stress” and connects to both ABCA1 and TXNRD2.

Probucol, an approved drug for hyperlipidemia, has been reported to inhibit ABCA1 activity [69] and increase SOD2 activity [70]. Both SOD2 and TXNRD2 are mitochondrial antioxidant stress enzymes [71], and mitochondrial dysfunction has been causally related to glaucoma [3]. Because probucol can ameliorate mitochondrial dysfunction [71], this observation raises the possibility that probucol could be used therapeutically as an OS suppressor for glaucoma. In fact, probucol protects against glutamate-induced cytotoxicity in a neuronal cell line [72] and, intriguingly, glutamate toxicity is one of the main pathogenic mechanisms of normal-tension glaucoma [73–76].

Other genes in the network could also be potential therapeutic targets for glaucoma-associated OS. For example, amyloid *β* (1–42), which is a proteolytic processing product of amyloid precursor protein (APP, underlined in green in Figure 2), is increased in the optic nerve head of monkeys and humans with glaucoma [77, 78]. Modulation of amyloid *β* aggregation can reduce apoptosis of retinal ganglion cells in a rat model of glaucoma [79]. OS and amyloid *β* aggregation exhibit reciprocal stimulation and induce neurodegeneration in both the brain and the retina [80]. In humans, an antibody against amyloid *β* reduces plaque formation and attenuates clinical decline in Alzheimer's disease [81]. Collectively, these findings suggest that amyloid *β* is a potential therapeutic target for glaucoma-associated OS.

Toll-like receptor 4 (TLR4, underlined in blue in Figure 2) is another potential target. Oxidized lipoprotein activates macrophages/microglia through TLR4 and promotes inflammation [82, 83]. Expressions of TLR4 and angiotensin II type 1 receptor (AGTR1) are increased in a mouse model of glaucoma, and an AGTR1 antagonist suppresses neurodegeneration in the mouse retina by inhibiting the TLR4-apoptosis signal-regulating kinase 1 pathway [84]. Modulators of TLR4 signaling have already been developed [85, 86], and the studies described here suggest that such modulators could be used to target OS in glaucoma.

### 2.3. An Integrative Approach towards Drug Discovery for AMD-Related OS

**Figure 3:**
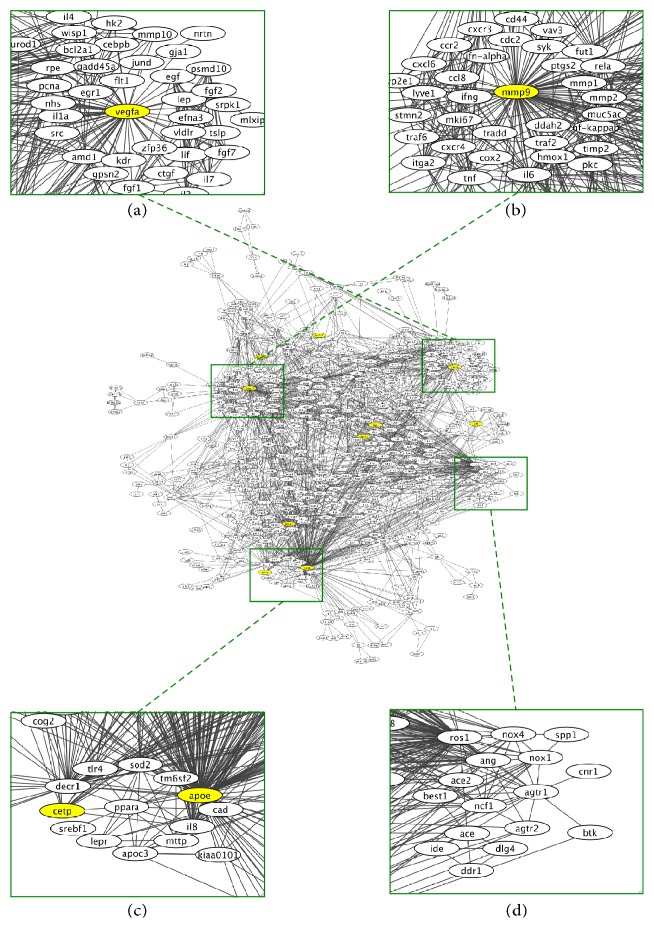
A network related to oxidative stress in age-related macular degeneration (AMD). An Agilent Literature Search was used as the basis for the network. We used 25 AMD-associated genes identified by GWAS as the target molecules and “oxidative stress” as the designated context in the Agilent Literature Search. The AMD-associated genes in the network are shown in yellow. The subnetworks containing potential therapeutic targets for AMD-related OS are enlarged in (a)–(d).

We applied the same approach to identify potential therapeutic targets for AMD-related OS. We used 25 AMD-associated genes identified by GWAS [87, 88] as the target molecules and “oxidative stress” as the designated context in an Agilent Literature Search. The resulting network (shown in Figure 3) contains ten AMD-associated genes (shown in yellow), including vascular endothelial growth factor A (VEGFA, Figure 3(a)) and matrix metallopeptidase 9 (MMP9, Figure 3(b)). Gene ontology analysis using DAVID [68] revealed that genes related to “response to oxidative stress” are significantly (*p* = 5.2 × 10^−16^) enriched in the network, suggesting that these ten AMD-associated genes are connected through genes related to OS.

OS is a major stimulator of VEGFA production and secretion by retinal pigment epithelial cells [89]. Notably, high VEGFA levels also increase oxidative damage, resulting in early degenerative changes in retinal pigment epithelial cells followed by neovascular AMD [90]. Intravitreal injection of anti-VEGF agents can slow the progression of neovascular AMD [12, 89], suggesting that VEGFA is one of the most important therapeutic targets for AMD-related OS.

OS also increases the expression of MMP9 [91] at an early stage of choroidal neovascularization (CNV) [92, 93]. The MMP inhibitors batimastat and marimastat reduce CNV when applied early in the process [93, 94], suggesting a potential therapeutic role for MMP9 inhibitors in AMD.

Other genes in the AMD-OS network may also be therapeutic targets. For example, peroxisome proliferator-activated receptor *α* (PPARA, Figure 3(c)) is the pharmacological target of fibrates such as fenofibrate, clofibrate, and bezafibrate [35]. PPARA is associated with both antioxidant and anti-inflammatory activities and has previously been suggested as a therapeutic target in AMD [95–97]. In fact, several clinical trials have revealed that fenofibrate can improve diabetic retinopathy [98, 99], which shares some pathophysiological mechanisms with AMD, including OS [100, 101]. These findings suggest that PPARA-activating drugs might have therapeutic utility for AMD.

Several components of the renin-angiotensin system, including angiotensin (ANG), angiotensin I converting enzyme (ACE), ACE2, and angiotensin II receptor type 1 and type 2 (AGTR1 and AGTR2), are shown in the subnetwork (Figure 3(d)). The renin-angiotensin system regulates various biological functions, including OS [102], and is hyperactivated in both AMD and diabetic retinopathy [103, 104]. Several clinical trials have demonstrated some efficacy of renin-angiotensin system inhibitors in slowing the progression of diabetic retinopathy [102, 105]. Thus, the renin-angiotensin system may also be a source of therapeutic targets for AMD-related OS.

## 3. Conclusion

Here, we provided an overview of some integrative approaches to drug discovery for OS in retinal diseases. We used two simple examples of glaucoma and AMD to illustrate how the approach can identify network hubs as potential therapeutic targets for these retinal diseases. The rapid advances in technology and increasing volume of multilevel omics data continue to create larger and more complex datasets to understand disease-associated biological networks and to build more extensive drug-target networks. Further progress in computational methodology combined with improved in vitro and in vivo disease models will facilitate the prioritization of therapeutic targets in the networks. The integration of multilevel omics data, computational approaches, and validated disease models will thus provide a strong foundation for deciphering the complex mechanisms of OS in retinal diseases and for discovering novel therapies with the greatest potential for efficacy in clinical trials.
